# Regeneration and defense: unveiling the molecular interplay in plants

**DOI:** 10.1111/nph.70171

**Published:** 2025-04-27

**Authors:** Dawei Xu, Li Yang

**Affiliations:** ^1^ Department of Plant Pathology, College of Agricultural & Environmental Sciences University of Georgia Athens GA 30602 USA; ^2^ Department of Plant Biology, Franklin College of Arts and Sciences University of Georgia Athens GA 30602 USA; ^3^ The Plant Center University of Georgia Athens GA 30602 USA

**Keywords:** defense‐growth crosstalk, disease resistance, immunity–regeneration balance, plant innate immunity, regeneration

## Abstract

In both plants and animals, tissue or organ regeneration typically follows wounding, which also activates defense responses against pathogenic microbes and herbivores. Both intrinsic and environmental cues guide the molecular decisions between regeneration and defense. In animal studies, extensive research has highlighted the role of various microbes – including pathogenic, commensal, and beneficial species – in influencing the signaling interplay between immunity and regeneration. Conversely, most plant regeneration studies are conducted under sterile conditions, which leaves a gap in our understanding of how plant innate immunity influences regeneration pathways. Recent findings have begun to elucidate the roles of key defense pathways in modulating plant regeneration and the crosstalk between these two processes. These studies also explore how microbes might influence the molecular choice between defense and regeneration in plants. This review examines the molecular mechanisms governing the balance between plant regeneration and innate immunity, with a focus on the emerging role of aging and microbial interactions in shaping these processes.

## Introduction

**Fig. 1:**
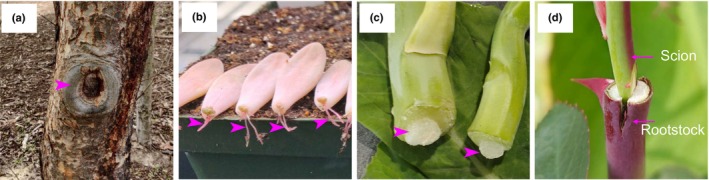
Examples of wound‐induced regeneration in plants. (a) Healed wound on a tree trunk showing a natural recovery process. Arrowhead points to the healed wound. (b) Detached succulent leaves regenerating adventitious roots. Arrowheads show adventitious roots. (c) Callus formed at cut stems of bok choy (*Brassica rapa* ssp. *chinensis*). Arrowheads indicate callus formation. (d) Grafted rose showing the scion (upper arrow) and the rootstock (lower arrow).

Plant cells exhibit a high level of developmental plasticity, enabling robust regenerative capabilities. Regeneration in plants is essential for the repair of mechanical damage, the replacement of injured organs, or recovery from herbivore feeding, all of which are vital for surviving in challenging environments. Plant regeneration also lays the foundation of critical agricultural techniques such as grafting, micropropagation, and the rooting of cuttings, as well as the production of transgenic plants (Chen *et al*., 2024). Most knowledge of the molecular mechanisms of plant regeneration comes from studies being conducted under aseptic conditions. However, in nature, regenerative processes occur in the presence of various microbes, including pathogens, commensal surface colonizers, and endophytes (Fig. 1). Wounds, which are a premise of regeneration, could be a direct consequence of herbivore feeding or mechanical damage and serve as entry points for pathogens, although many biotrophic pathogens may enter through natural openings like stomata or hydathodes, activating immunity through molecular recognition rather than wounding. An effective wound‐induced immune response may create an environment permissive to regeneration, whereas immune activation may also result in cell death and growth inhibition (He *et al*., 2022). Thus, a fine‐tuned molecular decision between defense and regeneration is critical for plant survival.

In animals, an inverse correlation between immune system complexity and regenerative capacity has been widely observed (Yun, 2015; Julier *et al*., 2017). Advanced immune systems in higher vertebrates often correlate with reduced regenerative capabilities. For example, primitive animals like flatworms and hydras can regenerate their entire body from cut pieces (Reddien & Alvarado, 2004; Vogg *et al*., 2019). Conversely, higher vertebrates such as mammals typically exhibit scar formation and reduced regenerative abilities following injury. This pattern extends to the ontogenesis of an organism. The ability to regenerate limbs decreases in axolotls after reaching their adult form (Vieira *et al*., 2020). The transition from scarless fetal wound healing to scar‐forming adult wound repair coincides with the maturation of the immune system in humans (Metcalfe & Ferguson, 2007). However, the mechanisms of how innate immunity interacts with regenerative processes in plants remain largely unexplored, presenting an important area for comparative research.

## Plant innate immunity: from receptors to hormonal network

Throughout their life cycles, plants are continuously exposed to microorganisms, so they have evolved a sophisticated immune system capable of quickly identifying foreign entities and effectively defending against potential pathogens. The plant immune response may include the establishment of physical barriers, the synthesis of antimicrobial compounds, a competition for nutrients with pathogens, and programmed cell death. The plant innate immune system incorporates the recognition of PATHOGEN‐ASSOCIATED MOLECULAR PATTERNS (PAMPs, also known as MICROBE‐ASSOCIATED MOLECULAR PATTERNS (MAMPs)) through PATTERN RECOGNITION RECEPTORS (PRRs) located on the cell surface, initiating PAMP‐TRIGGERED IMMUNITY (PTI) (Saijo *et al*., 2018). PAMPs are conserved molecules derived from microbes, including chitin from fungal cell walls, flagellin and elongation factor EF‐Tu from bacteria, and lipopolysaccharides from the outer membrane of Gram‐negative bacteria (Ngou *et al*., 2022). For example, the bacterial flagellin epitope flg22 is recognized by a surface‐localized receptor complex containing a leucine‐rich repeat serine/threonine protein kinase FLAGELLIN‐SENSITIVE 2 (FLS2) and its co‐receptor BRI1‐ASSOCIATED RECEPTOR KINASE (BAK1) (Sun *et al*., 2013). Other surface‐localized receptor‐like kinases (RLKs) or receptor‐like proteins (RLPs), critical for perceiving these microbial signals, orchestrate this rapid immune activation, highlighting their pivotal role in early plant defense (Huang & Joosten, 2025). Following the detection of PAMPs, PTI quickly activates, sometimes within seconds, downstream signaling events including REACTIVE OXYGEN SPECIES (ROS) burst, calcium influx, MITOGEN‐ACTIVATED PROTEIN KINASE (MAP kinase) cascade, and transcriptional reprogramming of the hormonal network (Saijo *et al*., 2018). Eventually, PTI leads to the synthesis of antimicrobial metabolites, fortification of cell walls, and depletion of nutrients and water to combat invading pathogens, serving as the first line of defense against pathogen invasion (Saijo *et al*., 2018). Some pathogens may counter these defensive measures by secreting effector proteins to suppress immune responses, hijack nutrients, or modify the host environment to their advantage. Plants use intracellular NUCLEOTIDE‐BINDING LEUCINE‐RICH REPEAT RECEPTORS (NLRs) to detect effectors directly or indirectly and activate a more targeted and robust response known as EFFECTOR‐TRIGGERED IMMUNITY (ETI) (Locci & Parker, 2024). ETI offers race‐specific resistance and often leads to programmed cell death as a strategy to limit pathogen multiplication. Recent research has revealed that PTI and ETI are interconnected and mutually reinforcing (Ngou *et al*., 2021; Yuan *et al*., 2021a,b). Integration of plant hormone signals into the transcriptional reprogramming network induced by both intracellular and cell surface receptors is a key step to fine‐tuning immune responses. Misfiring of the immune response may negatively affect plant growth, resulting in spontaneous cell death, stunted growth, and abnormal morphogenesis, so defense signaling is also highly integrated into the program of growth and development (van Butselaar & Van den Ackerveken, 2020). The interplay between phytohormones, such as the antagonistic interaction between salicylic acid (SA) and jasmonic acid (JA) fine‐tunes immune responses and ensures a balance between growth and defense mechanisms (Aerts *et al*., 2021). Beyond pathogenic interactions, microbes profoundly influence plant development and stress responses. Commensal and beneficial microbes also interact with the host immunity and influence the hormonal system directly by producing phytohormones and their mimics or indirectly via immune perception, potentially affecting regeneration processes. Certain endophytic bacteria may evade recognition by PRRs or inhibit PTI (Colaianni *et al*., 2021; Teixeira *et al*., 2021). Additionally, many microbes are capable of synthesizing phytohormones that directly influence plant growth (Nakano *et al*., 2022). This comprehensive signaling network underscores the complexity and effectiveness of plant immunity, providing robust and selective responses against various microbes in their environments.

## Divergent forms of regeneration with shared signaling components

Plant regeneration can be categorized into several types including tissue repair, *de novo* organogenesis, and somatic embryogenesis (Ikeuchi *et al*., 2016). Tissue repair refers to a plant's ability to heal damaged parts of its body, commonly seen after injuries in tissues such as root tips and stems. Grafting is used in agriculture to combine two desired traits by connecting plant parts from different species or varieties. It creates nontransgenic chimeras to gain better growth or higher resilience in various conditions, thus improving crops without genetic modification. *De novo* organogenesis involves the formation of new organs or even entire plants after injury, which is often seen in the formation of adventitious roots and shoots from a detached succulent leaf. Rooting from stem cutting, a form of *de novo* root regeneration (DNRR), is a routine agricultural practice for plant propagation. Somatic embryogenesis refers to the creation of a structure like a zygotic embryo from somatic cells, which then grows and develops into a new plant. Despite differences in the initial tissue type and the developmental trajectory that leads to new organs, these regeneration paths share several key steps. As a first step, wounding is an essential premise of regeneration. Wound‐derived signals, including phytohormones (e.g. JA) and damaged‐associated molecules (e.g. glutathione and ROS), play key roles in initiating the regeneration process (Zhang *et al*., 2019). While regeneration shares some signaling components with normal plant growth – such as auxin and cytokinin‐mediated hormonal cues – these two processes diverge in their triggers and developmental trajectory. Regular growth orchestrated by embryo‐originated meristems follows a genetically predetermined pattern to form various organs. By contrast, regeneration is an adaptive response often initiated by external stimuli, such as wounding or stress, and involves the reprogramming of differentiated cells into a pluripotent state. This dedifferentiation process, mediated by factors like WOUND INDUCED DEDIFFERENTIATION 1 (WIND1) or PLETHORA genes, allows plants to repair damage or replace lost tissues. In addition, the formation of callus, a group of highly proliferated and reprogrammed cells, is a shared step in several forms of plant regeneration. Dramatic reprogramming at the epigenetic and transcriptional levels allows cells to exit a resting cell cycle stage and initiate cell proliferation. Calli derived from various tissues may adopt a fate of lateral root primordia (Sugimoto *et al*., 2010). Depending on the hormonal cues and genetic regulation, pluripotent cells in the callus differentiate into specific cell types required for the regeneration of new organs or tissues. In this process, genes involved in establishing stem cell fates such as the WUS HOMEOBOX‐CONTAINING (WOX) protein family and the PLETHORA genes define shoots or roots meristem identity (Ikeuchi *et al*., 2019). Following that, organ morphogenesis defines the final step of regeneration to recreate an organ or whole plant.

Regeneration and immunity in plants are closely linked physiological processes, both initiated by wounding. Due to herbivore activity or microbial pathogen invasion, wounding not only triggers regeneration but also acts as an early signal for defense responses (Iwase *et al*., 2021). Early wound signals such as ROS burst, electrical signals, and changes in cytosolic calcium concentration play dual roles in regeneration and defense (Gilroy *et al*., 2016). Moreover, the JA pathway, while fundamental in regeneration, also activates defense‐related genes and stimulates the production of antimicrobial phytochemicals (Campos *et al*., 2014; Zhang *et al*., 2019; Zhou *et al*., 2019). Transcriptomic analyses have shown that early in the regeneration process, genes involved in the immune signaling response to pathogen‐derived molecules are activated, even in the absence of actual microbial invasion (Ikeuchi *et al*., 2017; Zhang *et al*., 2019; Liu *et al*., 2022). Importantly, genes that are pivotal for regeneration may also function in defense responses. Thus, following initial wounding, plants must navigate a molecular decision to prioritize either a regeneration or defense pathway. Here, we summarize recent advances in understanding the immunity–regeneration crosstalk and key players in this process (Table 1).

**Table 1:** Genes with dual functions in regeneration and immunity.

Gene name	Role in immunity	Role in regeneration
*WIND1* (AT1G78080)	Enhances pathogen response and resistance to *Pseudomonas syringae* DC3000 (Iwase *et al*., 2021; Ribeiro *et al*., 2024)	Promotes callus formation and regeneration at wound sites by activating ESR1 (Iwase *et al*., 2011, 2013, 2015, 2017, 2018)
*ICS1* (AT1G74710)	Essential for SA biosynthesis, especially in defense responses (Wildermuth *et al*., 2001)	Loss‐of‐function mutant enhances callus formation and *de novo* root regeneration (Tran *et al*., 2023a,b)
*NPR1* (AT1G64280)	Binds to SA and promotes SA‐induced immune responses, functions as a transcriptional co‐activator in plant defense (Kumar *et al*., 2022)	Required for SA‐mediated suppression of *de novo* root regeneration (Hernández‐Coronado *et al*., 2022)
*NPR3* (AT5G45110) *NPR4* (AT4G19660)	Function as transcriptional co‐repressors in plant defense, inhibiting SA‐induced immune responses (Ding *et al*., 2018)	Positively regulates *de novo* root regeneration (Tran *et al*., 2023a)
*GLR1.2* (AT5G48400) *GLR1.4* (AT3G07520) *GLR2.2* (AT2G24720) *GLR3.3* (AT1G42540)	GLR3.3 is required for oligogalacturonides and GSH‐induced defense response (Li *et al*., 2013; Manzoor *et al*., 2013; Hernández‐Coronado *et al*., 2022)	Suppresses callus formation and regeneration (Hernández‐Coronado *et al*., 2022)
*SPL2* (AT5G43270) *SPL10* (AT1G27370) *SPL11* (AT1G27360)	Promotes age‐related resistance in Arabidopsis by enhancing SA‐mediated immunity. SPL10 directly activates PAD4 (Hu *et al*., 2023)	Suppresses root regeneration in aging plants by inhibiting wound‐induced auxin biosynthesis through direct repression of AP2/ERF transcription factors (Ye *et al*., 2020)
*FER* (AT3G51550)	Enhances plant immunity by destabilizing MYC2 to inhibit JA signaling (Guo *et al*., 2018)	Promotes root regeneration by interacting with TPL/TPRs to regulate regeneration‐related genes (Xie *et al*., 2022)
*PORK1* (Solyc03g123860)	Required for systemin‐activated defense pathways and JA signaling, enhancing resistance to pathogens and herbivores (Xu *et al*., 2018)	Promotes plant regeneration by perceiving the REF1 signal and activating SlWIND1 (Yang *et al*., 2024)
*REF1* (Solyc04g072310)	Facilitates expression of defense genes in response to wounding (Yang *et al*., 2024)	Perceived by PORK1 to enhance regeneration through slWIND1 activation (Yang *et al*., 2024)

## Salicylic acid integrates biotic and abiotic cues to suppress regeneration signaling

Salicylic acid is important in coordinating resistance to biotrophic, hemi‐biotrophic, and necrotrophic pathogens in different plant species, while also engaging in complex crosstalk with abiotic stress responses and other hormonal pathways, such as JA, to modulate plant defense and physiology (De Vleesschauwer *et al*., 2013). These pathogens either fully rely on living (biotrophic) or dead (necrotrophic) plant tissues for nutrients, or can switch between feeding on living or dead tissues during their life cycle (hemi‐biotrophic). Our current understanding of the SA signaling pathway largely derives from its role in plant immunity in Arabidopsis. Pathogen‐induced SA is primarily synthesized through the ISOCHORISMATE (IC) pathway. The Arabidopsis genome encodes two chloroplast‐localized isochorismate synthases, ISOCHORISMATE SYNTHASE 1 (ICS1) and ISOCHORISMATE SYNTHASE 2 (ICS2). ENHANCED DISEASE SUSCEPTIBILITY 5 (EDS5), a MULTIDRUG AND TOXIC COMPOUND EXTRUSION (MATE) transporter, facilitates the transport of IC from plastids to the cytosol. In the cytosol, the enzyme AVRPPHB SUSCEPTIBLE3 (PBS3) catalyzes the conjugation of IC and glutamate to produce SA (Rekhter *et al*., 2019). Additionally, PHENYLALANINE AMMONIA‐LYASE (PAL) converts phenylalanine to *trans*‐cinnamic acid, offering an alternative SA biosynthesis route. SA is recognized by members in the NONEXPRESSER OF PATHOGENESIS RELATED (NPR) protein family, which possess a BTB (BROAD‐COMPLEX, TRAMTRACK AND BRIC A BRAC)/POZ (POXVIRUS AND ZINC FINGER) domain and an ankyrin repeat domain (Rochon *et al*., 2006). NPR1 functions as a transcriptional co‐activator that collaborates with TGA transcription factors to regulate SA‐responsive genes, while NPR3 and NPR4 can repress these defenses either independently or by promoting NPR1 degradation (Zhou *et al*., 2023). NPR5 and NPR6, also known as BLADE‐ON‐PETIOLE 2 and 1 (BOP2 and BOP1), lack critical amino acids for SA‐binding, so their roles in SA‐dependent responses are not clear yet (Zhou *et al*., 2023). The effects of SA or its analogs on wound‐induced tissue regeneration remain varied across different studies. Some research indicates that SA pre‐treatment can enhance shoot regeneration in grapevine (*Vitis* spp.), *in vitro* shoot organogenesis in *Cucumis melo*, and somatic embryogenesis in carrot (Roustan *et al*., 1990; Shetty *et al*., 1992; Pathirana *et al*., 2016). Conversely, other studies report that SA negatively impacts regeneration, such as inhibiting adventitious root formation in mung bean hypocotyl cuttings and disrupting IAA‐induced adventitious root formation in apple micro‐cuttings by increasing IAA decarboxylation (De Klerk *et al*., 2011; Yang *et al*., 2013).

Recent research in Arabidopsis has elucidated the molecular function of SA in integrating biotic and abiotic signals to suppress regeneration. SA may operate downstream of glutamate receptors, a family of cation‐permeable ion channels, to inhibit *de novo* organogenesis and callus formation (Hernández‐Coronado *et al*., 2022). Plant GLUTAMATE RECEPTOR‐LIKE (GLR) homologs, similar to their animal counterparts involved in neurotransmission, are crucial in managing Ca^2+^ influx through the plasma membrane, influencing various physiological processes. These receptors are essential in launching the plant immune response to PAMPs and other elicitors, such as oligogalacturonides (OGs) derived from cell wall degradation (Manzoor *et al*., 2013). Genetic and pharmacological studies have linked GLR functionality to resistance against pathogens such as *Hyaloperonospora arabidopsidis* (Manzoor *et al*., 2013). Interestingly, the Arabidopsis quadruple mutant *glr1.2/1.4/2.2/3.3* showed enhanced regeneration efficiency. GLR‐mediated suppression of callus formation or DNRR requires the endogenous SA perception and biogenesis (Hernández‐Coronado *et al*., 2022). Thus, the GLR–SA cascade coordinates a defense–regeneration trade‐off in plants. Furthermore, SA is also involved in relaying hypoxia stress to inhibit regeneration (Koo *et al*., 2024). Under hypoxic conditions created by limited gas exchange in developing calli, the hypoxia‐activated RELATED TO APETALA 2.12 (RAP2.12) directly binds to the promoter of the ICS1 gene, activating SA biosynthesis, which in turn hinders the acquisition of pluripotency and *de novo* shoot regeneration in calli (Koo *et al*., 2024). These findings suggest that SA integrates multiple stress signals affecting shoot regeneration from callus.

**Fig. 2:**
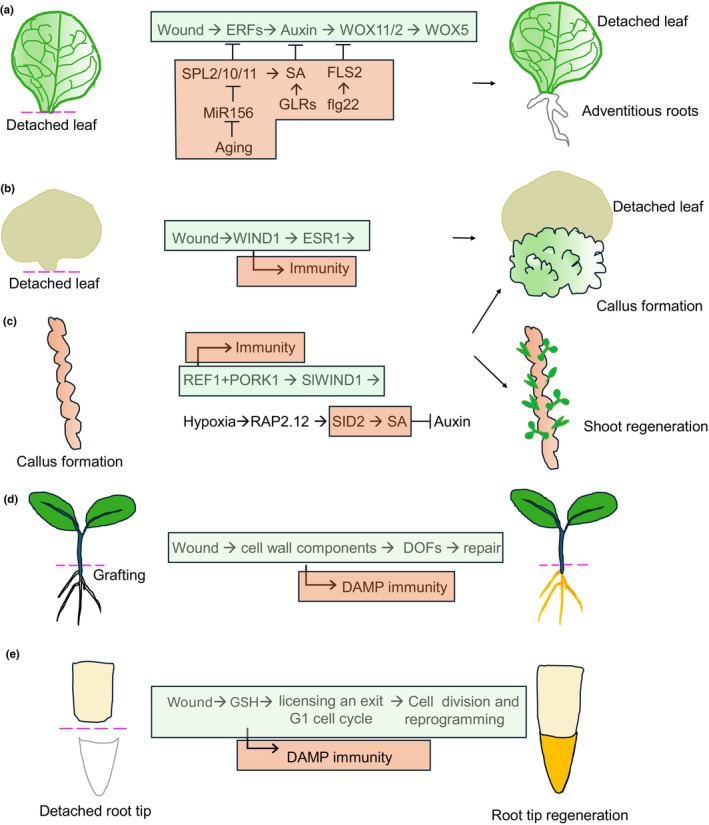
Schematic representation of crosstalk between immunity and various forms of plant regeneration in *Arabidopsis thaliana* or tomato (*Solanum lycopersicum*). (a) Pathogen‐associated molecular patterns (PAMP)‐triggered immunity, salicylic acid (SA) signaling, and aging collaboratively inhibit *de novo* root regeneration (DNRR). (b) WIND1 promotes defense and callus formation after wounding. (c) REF1, as a wound‐induced signal, activates immunity and *de novo* shoot regeneration in tomato. SA relays hypoxia stress to inhibit *de novo* shoot regeneration. (d) Degraded cell wall components activate tissue repair via DOFs and DAMP‐induced immunity. (e) GSH released from damaged cells may trigger neighboring cells to exit G1 phase, enabling cell division and reprogramming during root tip regeneration. GSH may also activate immunity as a damage signal. DAMP, DAMAGE‐ASSOCIATED MOLECULAR PATTERNS; DOF, DNA BINDING WITH ONE FINGER; ESR1, ENHANCER OF SHOOT REGENERATION 1; FLS, Flagellin Sensing 2; GSH, glutathione; PORK1, PEPR1/2 ORTHOLOG RECEPTOR‐LIKE KINASE 1; RAP2.12, RELATED TO APETALA 2.12; REF1, REGENERATION FACTOR1; SID2, SA induction‐deficient 2; SlWIND1, *Solanum lycopersicum* WOUND INDUCED DEDIFFERENTIATION 1; WIND1, WOUND INDUCED DEDIFFERENTIATION 1. Arrows indicate the promotion of a process; blocking bar symbols represent the inhibition of a process.

Components in the SA‐mediated defense are differentially required for plant regeneration. The ICS1 knockout mutant *SA induction‐deficient 2* (*sid2*), deficient in SA biosynthesis, exhibited enhanced adventitious root formation and callus formation capabilities (Hernández‐Coronado *et al*., 2022; Tran *et al*., 2023a). NPR1 and NPR3/4 proteins show divergent roles in regeneration. NPR3/4 positively regulates DNRR (Tran *et al*., 2023a), while NPR1 may be dispensable or play a negative role (Hernández‐Coronado *et al*., 2022). Although the double mutants *npr5/6* enhanced DNRR, it is unclear whether NPR5/6 regulates regeneration via interacting with NPR1/3/4 or competing for shared protein partners (Tran *et al*., 2023a). The CALMODULIN BINDING PROTEIN 60G (CBP60g), a transcriptional target of NPR3/4, is required for SA‐mediated defense response, but does not alter DNRR in its mutant form (Tran *et al*., 2023a). Additionally, SA has been shown to partially suppress adventitious root formation by inhibiting the expression of auxin transport genes, impeding the establishment of an auxin maximum at cutting sites (Tran *et al*., 2023a). These observations suggest a divergence in SA‐mediated pathways upstream of CBP60g, feeding into defense and regeneration. Transient treatment with SA signaling inhibitors, such as 4‐phenyl‐2‐{[3‐(trifluoromethyl)‐aniline]methylidene}cyclohexane‐1,3‐dione (PAMD) or inhibitors of glutamate‐like receptor 6‐Cyano‐7‐nitroquinoxaline‐2,3‐dione (CNQX), has been shown to enhance regeneration efficiency in both dicots and monocots, offering a promising tool to improve regeneration in recalcitrant species (Hernández‐Coronado *et al*., 2022). It is noteworthy that PAMD enhances regeneration and inhibits SA‐mediated defense signaling, which may compromise disease resistance when pathogens are present. Identifying the components that are specifically required for SA‐mediated suppression of regeneration, but not defense, would be useful to decouple these two processes in future genetic engineering to achieve high regeneration capacity without compromising defense. These insights from different regeneration systems, including callus formation from leaves and root regeneration, underscore the complex interplay between SA signaling and plant regeneration processes (Fig. 2). However, their generalizability beyond roots – such as to shoot regeneration or somatic embryogenesis – may vary due to tissue‐specific hormonal interactions and developmental cues, calling for further investigation.

Notably, the basal level and signaling components of SA pathways are differentially regulated across plant species (Ullah *et al*., 2023). Redox modification on key cysteine residues is critical for AtNPR1's role in activating SA signaling. However, a key residue in AtNPR1 for thiol–disulfide exchange (e.g. Cys^156^) is restricted to a small group of plants belonging to Brassicaceae (Ullah *et al*., 2023). The basal levels of SA in rice and Populus could be 10‐ to 20‐fold higher than that in Arabidopsis (De Vleesschauwer *et al*., 2013; Ullah *et al*., 2023). Although AtNPR1 is a master receptor of SA in Arabidopsis, it is not solely required for SA response in rice (Shimono *et al*., 2007; Matsushita *et al*., 2013). Even different Arabidopsis accessions possess variable levels of SA, which contribute to variations in defense response (Van Leeuwen *et al*., 2007; Velásquez *et al*., 2017). These variations may profoundly influence the outcomes of immunity–regeneration crosstalk. Further exploration is needed to fully understand the SA‐mediated immunity–regeneration nexus in different plants.

## Wound signaling activates both defense and regeneration

Wound‐derived signals in plants can simultaneously initiate defense and regeneration pathways, though their molecular divergence remains poorly understood. Jasmonic acid plays a central role in transmitting wound signals and regulating regeneration. We direct readers to a recent outstanding review on the roles of JA in plant regeneration (Zhang *et al*., 2023). Here we discuss recent studies that have shed light on how damage‐induced signals balance these two processes. The APETALA2/ETHYLENE RESPONSE FACTOR (AP2/ERF) transcription factor WIND1 positively regulates both regeneration and defense (Iwase *et al*., 2021). WIND1, together with its homologues (WIND2, 3, and 4), promotes cell dedifferentiation at wound sites in Arabidopsis, rapeseed, tomato, and tobacco (Iwase *et al*., 2011, 2013). In Arabidopsis, WIND1 operates through the B‐type ARABIDOPSIS RESPONSE REGULATOR (ARR)‐mediated cytokinin response pathway and enhances shoot regeneration by activating ENHANCER OF SHOOT REGENERATION1 (ESR1) (Iwase *et al*., 2017). This activation suggests a direct linkage between wound signaling and regenerative processes. Moreover, transcriptome analyses following WIND1 induction show significant changes in genes involved in cellular reprogramming, vascular development, and pathogen response (Iwase *et al*., 2021). The *wind1* mutant showed enhanced susceptibility to the bacterial pathogen *Pseudomonas syringae* DC3000 (Iwase *et al*., 2021). These insights highlight WIND1's pivotal role in orchestrating multifaceted physiological responses to tissue injury, although the detailed mechanism for WIND1‐mediated defense remains to be elucidated. In tomato, REGENERATION FACTOR1 (REF1) synergizes with systemin, a peptide hormone, to boost the activation of defense genes both locally at the wound site and systemically across the plant. The JA biosynthetic pathway is essential for transmitting the systemin signal to generate a long‐distance signal (Li *et al*., 2002). Beyond its role in defense, REF1 also serves as a wound peptide signal that triggers regeneration processes (Yang *et al*., 2024). This function is mediated through its recognition by the PEPR1/2 ORTHOLOG RECEPTOR‐LIKE KINASE 1 (PORK1) receptor. The interaction between REF1 and PORK1 activates SlWIND1 to enhance regenerative processes. Moreover, the exogenous application of REF1 peptides significantly enhances the regeneration efficiency in recalcitrant varieties of tomato, soybean, wheat, and maize, providing a novel approach to improve crop transformation (Yang *et al*., 2024). The dual roles of REF1 and WIND1 in both regeneration and defense suggest the existence of an early signaling stage that transduces wound signals to these processes before their pathways diverge.

**Fig. 3:**
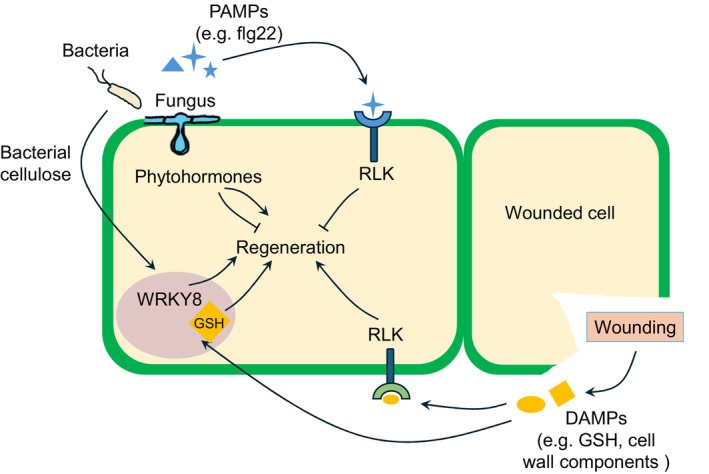
Potential microbial influence on plant regeneration. Microbes, such as bacteria and fungi, may influence regeneration by producing or degrading phytohormones. Additionally, pathogen‐associated molecular patterns (PAMPs), such as the 22‐amino‐acid epitope of bacterial flagellin (flg22) and bacterial cellulose, could be perceived as environmental cues that inhibit or promote regeneration. Cell wall debris or intracellular contents, such as glutathione (GSH) released from damaged cells due to pathogen infection, may be recognized by neighboring cells, promoting regeneration. Receptor‐like kinases (RLKs), including Flagellin Sensing 2 (FLS2), Somatic Embryogenesis Receptor‐like Kinases (SERKs), and FERONIA receptor‐like kinase (FER), perceive these signals and mediate downstream responses. Arrows indicate the promotion of a process; blocking bar symbols represent the inhibition of a process. DAMP, DAMAGE‐ASSOCIATED MOLECULAR PATTERNS; WRKY8, WRKY DNA‐BINDING PROTEIN 8.

DAMAGE‐ASSOCIATED MOLECULAR PATTERNS (DAMPs) are released by plant cells upon stress, damage, or cell death, serving as danger signals that activate the immune system (Tanaka & Heil, 2021). Molecules such as ATP, oligosaccharides, NAD(P), glutathione (GSH), and specialized peptides can act as DAMPs, playing crucial roles in defending against infections and facilitating tissue repair (Tanaka & Heil, 2021). In animals, HIGH MOBILITY GROUP BOX 1 (HMGB1) is a nonhistone nuclear protein and can be passively released as a classic DAMP (Chen *et al*., 2022). ATP may act as a chemoattractant for immune cells, delivering a ‘find‐me’ signal that is recognized by macrophages (Klune *et al*., 2008; Elliott *et al*., 2009). While these examples illustrate how damage‐associated signals function in animal systems, a parallel phenomenon occurs in plants. For instance, it is established that oligosaccharide, such as OGs and mixed‐linked β‐1,3/1,4‐glucans (MLGs) released from damaged cell walls serves as signals to activate plant immune responses. However, the link between cell wall damage and regeneration in plants has only been revealed recently. Wounding triggers the expression of several DNA BINDING WITH ONE FINGER (DOF) transcription factors, including HIGH CAMBIAL ACTIVITY2 (HCA2) and TARGET OF MONOPTEROS6 (TMO6) (Zhang *et al*., 2022). Their quadruple *hca2*, *tmo6*, *dof2.1*, and *dof6* mutant reduced various forms of regeneration, including callus formation, tissue attachment, and vascular regeneration in response to wounding. Pharmaceutical and genetic modifications to the cellulose or pectin matrix activates TMO6 and HCA2, indicating that cell wall damage acts as a wound‐associated signal promoting tissue regeneration via these transcriptional factors (Zhang *et al*., 2022). The involvement of these DOF transcription factors in wound‐induced defense responses remains unclear; however, their interaction with MYC2, a key transcription factor that regulates JA‐responsive genes via the MYC2‐DOF2.1 feedback loop, indicates a potential dual role in both defense and regeneration (Zhuo *et al*., 2020). FERONIA (FER), a receptor kinase, may be another link connecting immunity and regeneration by sensing cell wall integrity. FER inhibits JA‐ (or coronatine) induced signaling by phosphorylating and destabilizing MYC2 (Guo *et al*., 2018). Interestingly, *fer* mutants also showed enhanced root tip regeneration (Xie *et al*., 2022). Although the extracellular domain of FER can associate with pectin, a cell wall component (Duan *et al*., 2020; Tang *et al*., 2022), it is not clear whether its function in immunity and regeneration relies on its association with pectin. Release of cytosolic components may also serve as a damage signal to activate immune response. Treatment of GSH, a tripeptide in cells, triggers typical immune responses and inhibits pathogen propagation, which is dependent on GLR3.3 (Li *et al*., 2013). In addition to its role as a DAMP for activating immune response, GSH from injured tissues also prompts cells near the wound to exit the G1 phase of the cell cycle, facilitating rapid cell division and reprogramming (Lee *et al*., 2025). This process is evidenced by a nuclear increase of GSH preceding synchronized entry into the S phase. Cells with a shortened G1 phase undergo cell fate reprogramming more rapidly. These studies provide a direct link between tissue damage and regeneration (Figs 2, 3). Remarkably, more than two dozen DAMPs and their respective receptors have been characterized for their function in activating immune response (Tanaka & Heil, 2021). It would be interesting to know how many of them contribute to wound‐induced regeneration and whether they have distinct functions in influencing regeneration.

## Role of microbial recognition in plant regeneration

The influence of plant‐associated microbes on regeneration processes presents several unique characteristics. Initially, these interactions are heavily influenced by the wound response, which may alter the typical host–microbe dynamics. For instance, wounding is necessary for opportunistic *Xanthomonas* pathogens within the plant microbiota to be pathogenic and degrade Arabidopsis leaf tissue (Pfeilmeier *et al*., 2024). Moreover, regenerated tissues such as calli or *de novo* shoots and roots create new ecological niches for microbial colonization. The interaction between microbes and host cells in these newly regenerated tissues could differ from those in mature tissues due to variations in immune responses and the tissue‐specific expression of immune‐related genes, such as the compromised SA signaling in young leaves (Hu *et al*., 2024). Additionally, cells undergoing fate transitions during regeneration are typically located beneath the surface of the callus (Zhai & Xu, 2021), resulting in unique immune signaling that differs from cells directly interacting with microbes. Despite their importance to plant immunity and regeneration, the impact of plant microbiota and the contribution of individual members on regeneration remain unclear, representing a significant gap in current regeneration studies.

In many plant regeneration systems, such as tissue culture and micropropagation, microbial presence is generally considered as contamination and is actively eliminated through methods like re‐culturing or antibiotics (Leifert & Cassells, 2001). However, biotization, defined as the metabolic response of *in vitro*‐grown plant material to microbial inoculants, may induce beneficial changes in plant regeneration (del Rosario Espinoza‐Mellado *et al*., 2021). This phenomenon can occur throughout all stages of *in vitro* propagation and may exert direct or indirect effects on plant growth via providing essential nutrients, enhancing the plant's ability to withstand adverse conditions, or providing protection against pathogens through the production of antibiotics (del Rosario Espinoza‐Mellado *et al*., 2021). Inoculation with *Paenibacillus* spp. increases root length and microcutting numbers in poplar (Vaitiekūnaitė *et al*., 2021). Similarly, biotization of banana plantlets with *Methylobacterium salsuginis* improved their survival and growth under both glasshouse and outdoor conditions (Pushpakanth *et al*., 2021). Despite beneficial effects of some microbes on tissue regeneration, certain conserved microbial patterns, such as flg22, a peptide derived from bacterial flagellin, inhibit regeneration from Arabidopsis leaf explants (Tran *et al*., 2023b). This inhibition, which is dependent on the immune receptor FLS2, occurs independently of SA, but is mitigated in mutants with elevated auxin levels (Tran *et al*., 2023b). In addition, SOMATIC EMBRYOGENESIS RECEPTOR‐LIKE KINASE 1 (SERK1) is a positive regulator of somatic embryogenesis, influencing plant regeneration processes across various species, including *Coffea canephora*, *Arabidopsis thaliana*, and *Oryza sativa* (Hu *et al*., 2005; Pérez‐Pascual *et al*., 2018). Overexpression of the *C. canephora* SERK1 homolog in transgenic embryogenic explants induced the expression of genes associated with auxin metabolism and the activation of early‐stage homeotic genes such as *WUSCHEL* (*WUS*), *BABY BOOM* (*BBM*), and *AGAMOUS‐LIKE 15* (*AGL15*) (Pérez‐Pascual *et al*., 2018). Ectopic expression of SERK1 significantly enhances the efficiency of somatic embryogenesis (Hu *et al*., 2005; Pérez‐Pascual *et al*., 2018). Although SERK1's contribution to plant immunity is unclear, it interacts with EFR‐ and FLS2‐containing PRR complexes potentially serving as a signaling node relaying immune signal to regeneration process (Roux *et al*., 2011). It is also noteworthy that BAK1 (also known as SERK3), a co‐receptor involved in recognizing many microbe‐derived molecules, plays a key role in transducing immune signals (Chinchilla *et al*., 2009). These observations suggest that interactions between SERK family members and other surface‐localized receptors are crucial for interpreting biotic cues from the environment and deciding between launching defense responses or activating regenerative pathways. It would be intriguing to further dissect the function of SERKs in coordinating defense‐regeneration balance in the presence of microbes. A recent study by Ruiz‐Solaní *et al*. showed that bacterial cellulose, but not plant cellulose or agarose, triggers wound regeneration in *Nicotiana benthamiana* and Arabidopsis leaves. This bacterial cellulose‐specific process activates cytokinin together with a sustained burst of ROS. In this process, WRKY DNA‐BINDING PROTEIN 8 (WRKY8) transcription factor regulates ROS balance and superoxide accumulation to support cell proliferation after wounding. These findings highlight a role of bacterial‐derived molecule (e.g. bacterial cellulose) to drive plant regeneration alongside with activating defense (Ruiz‐Solani *et al*., 2025). Thus, the impact of a specific microbe on plant regeneration is a consequence of complex interactions. While activated PTI may inhibit regeneration, specific microbial actions could alternatively promote regeneration pathways (Fig. 3).

## Immunity links aging and regeneration in plants

Like animals, plants' regeneration capacities decline as they age. Recent research suggests that the age‐associated decline of regeneration is intimately linked with enhanced innate immunity. Disruption of the SA pathway, either through chemical conversion of SA to catechol in NahG transgenic plants or by genetic mutation in NPR1, blocked the age‐related decline in DNRR (Hernández‐Coronado *et al*., 2022; Tran *et al*., 2023a), suggesting that an increase in SA‐mediated suppression of regeneration occurs during plant aging. Indeed, a gain of SA response was observed during both shoot maturation and leaf expansion (Hu *et al*., 2023, 2024). Additionally, leaf maturation is associated with increased activity of ETHYLENE‐INSENSITIVE 3 (EIN3), an ethylene response factor that suppresses the transcription of WOX11 and WOX5, both necessary for DNRR (Liu *et al*., 2014; Li *et al*., 2020). During the vegetative phase change from juvenile to adult stages, three microRNA 156 (miR156)‐targeted SQUAMOSA PROMOTER BINDING PROTEIN‐LIKE (SPL) transcription factors – SPL2, SPL10, and SPL11 – suppress regeneration and enhance immunity (Ye *et al*., 2020; Hu *et al*., 2023). Due to the temporal reduction of miR156 accumulation in shoot maturation, SPL2/10/11 are preferentially accumulated in adult leaves where they inhibit regeneration by directly binding to and repressing the promoters of AP2/ERF transcription factors, such as ABSCISIC ACID REPRESSOR1 (ABR1) and ETHYLENE RESPONSE FACTOR 109 (ERF109), which are involved in wound signaling and auxin biosynthesis (Ye *et al*., 2020). Interestingly, SPL10 also binds to the promoter of PHYTOALEXIN DEFICIENT 4 (PAD4), a critical component of the SA signaling pathway and ETI, leading to enhanced basal resistance in adult plants (Hu *et al*., 2023). Thus, the SPL2/10/11 genes integrate a developmental clock to balance age‐dependent regeneration and immunity. On the other hand, JA response progressively decays in Arabidopsis during shoot maturation. This age‐dependent process is regulated by another miR156‐targeted SPL gene, SPL9 (Mao *et al*., 2017). As SPL9 levels gradually increase during shoot maturation, it interacts with and stabilizes JASMONATE‐ZIM DOMAIN proteins (JAZs), which are suppressors of JA signaling, leading to attenuation of the JA response (Mao *et al*., 2017). Wound‐induced JA response may activate auxin biosynthesis through ERF109 (Zhang *et al*., 2019). Thus, the miR156‐SPL module has multifaceted roles in regulating age‐dependent regeneration and immunity. Age‐dependent decline of regeneration is a bottleneck in plant propagation and breeding (Thomas, 2011). These discoveries provide valuable targets for future genetic manipulations to decouple plant aging and regeneration decline.

## Perspective

Despite significant advancements in understanding the molecular mechanisms that mediate the crosstalk between plant immunity and regeneration, several key questions remain unresolved. First, what are the molecular signals that prioritize defense or regeneration after wounding? Is there a temporal or spatial separation of these two processes? Rapid development in high‐resolution, single‐cell techniques may allow scientists to investigate fundamental questions about how distinct cell types, particularly pluripotent cells, interpret and respond to immune signals. Moreover, the immune response is profoundly shaped by the presence of various microbes, which have developed complex strategies to influence specific immune pathways through effectors, toxins, and phytohormones. It is crucial to decipher how environmental microbes and endophytic communities affect regeneration capacity and how these microbes colonize newly formed tissues. Furthermore, both defense and regeneration are tightly regulated by spatiotemporal patterns. Tissues exhibit a concentration‐dependent response to phytohormones, so molecular interactions observed in the root may not necessarily apply to the shoot. Other plant hormones (e.g. auxin, gibberellic acids and abscisic acids) and nutrient availability also influence both defense and regeneration. Although the molecular details of how these factors directly link regeneration and defense remain limited, further research is essential to explore the complex hormonal network that deciphers intrinsic and environmental cues to balance regeneration and defense. Finally, various forms of regeneration, such as grafting and rooting from cuttings, are essential to modern agriculture. In particular, tissue culture is a crucial step in most plant transformation systems. Over many decades, crop breeding has been focused on disease resistance as a prime trait. Sometimes, regeneration‐dependent methods like grafting and cuttings are used to breed elite varieties. While breeding strategies for most row crops do not involve regeneration processes. This raises a question: Has the focus on disease resistance in breeding inadvertently constrained the regeneration efficiency of some elite varieties? In parallel with the advances in plant genome editing, a more balanced approach that considers regeneration capacity should be adopted for future breeding strategies.

## Competing interests

None declared.

## Author contributions

DX and LY collected data and wrote the manuscript.

## Disclaimer

The New Phytologist Foundation remains neutral with regard to jurisdictional claims in maps and in any institutional affiliations.
